# METTL3 overexpression aggravates LPS-induced cellular inflammation in mouse intestinal epithelial cells and DSS-induced IBD in mice

**DOI:** 10.1038/s41420-022-00849-1

**Published:** 2022-02-14

**Authors:** Lichao Yang, Guotao Wu, Qiang Wu, Liangxin Peng, Lianwen Yuan

**Affiliations:** Department of Geriatric Surgery, The Second Xiangya Hospital, Central South University, Changsha, Hunan China

**Keywords:** Genetics, Gastrointestinal diseases, Immunological disorders

## Abstract

The inflammatory bowel diseases (IBD), including Crohn’s disease (CD) and ulcerative colitis (UC), are chronic inflammatory disorders of the intestine. Dysregulated cytokine secretion and signal transduction mechanisms via intestinal epithelial cells are involved in IBD pathogenesis, in which the transcription factor NF-κB plays a critical role. In this study, METTL3, which plays a key role in inflammation regulation, has been recognized significantly up-regulated in IBD samples, DSS-induced IBD mice, and LPS-treated MODE-K cells. Within LPS-treated MODE-K cells, METTL3 knockdown promoted cell viability, inhibited cell apoptosis, decreased apoptotic caspase3/9 cleavage, and decreased the levels of proinflammatory cytokines (IL-1β, TNF-α, IL-6, and IL-18) and inflammatory enzymes (COX-2 and iNOS). Under the same conditions, METTL3 knockdown inhibited, whereas METTL3 overexpression promoted p65 phosphorylation in MODE-K cells; NF-κB inhibitor JSH-23 partially abolished the promotive effects of METTL3 overexpression upon p65 phosphorylation. Consistently, the effects of METTL3 overexpression upon LPS-stimulated MODE-K cells were partially abolished by JSH-23. Lastly, METTL3 knockdown in DSS-induced IBD mice significantly ameliorated DSS-induced IBD and inhibited DSS-induced p65 phosphorylation. In conclusion, METTL3 overexpression aggravates LPS-induced cellular inflammation in mouse intestinal epithelial cells and DSS-induced IBD in mice. The NF-κB signaling might be involved, and the regulatory mechanism remains to be investigated in our future study.

## Introduction

The inflammatory bowel diseases (IBDs), including Crohn’s disease (CD) and ulcerative colitis (UC) [1, 2], are chronic inflammatory disorders of the intestine. The clinical course of IBD is marked by periods of symptomatic relapse and remission that are characterized by diarrhea and intestinal bleeding, resulting in epithelial barrier disruption and epithelial ulceration formation [3], with extensive damage to intestinal structures [4]. IBD is gradually becoming a global disease with rapidly increasing incidence and prevalence [5]. Up to now, its etiology and pathogenesis have not yet been clarified. Nevertheless, the current understanding of IBD pathogenesis implicates a complex crosstalk between host genetics, host immunity, microbiome, and environmental exposures [6].

Dysregulated cytokine secretion and signal transduction mechanisms via intestinal epithelial cells, lymphocytes, and macrophages are involved in IBD pathogenesis, and the transcription factor NF-κB has been shown to be a main regulatory component within this complex situation [7]. NF-κB expression and activation were shown to be markedly induced within IBD patients’ inflamed gut. In particular, the enhancement of NF-κB p65 exists in macrophages and epithelial cells isolated from inflamed gut samples of patients with IBD [7]. NF-κB activation under this condition has been analyzed via immunofluorescence staining of activated p65 in biopsies, which clearly indicated both the activation and the expression of NF-κB within the mucosal macrophages and epithelial cells of patients with IBD Notably, a significant correlation between activation NF-κB number and intestinal inflammation severity has been observed [8]. Considering the wide range of NF-κB-regulated genes, it’s necessary to evaluate the cell-specific roles of NF-κB involved in IBD pathogenesis and identify events that might cause the NF-κB signaling deregulation.

The most prevalent internal modification on eukaryotic mRNA is m6A [9]. m6A modification, a reversible dynamic process, is believed to be the methylation of the sixth position of nitrogen atom of adenosine [10]. As compared to a variety of chemical mechanisms, m6A modification has attracted much attention in its potential to dynamically and reversibly regulate gene expression at a posttranscriptional level. It can interact with various RNAs and signaling pathways, making it exert a critical effect on the progression of diseases. There is mounting evidence that m6A methylation, as a crucial mode of posttranscriptional gene regulation, is involved in the pathogenesis of IBD [11]. For instance, the absence of a component of RNA m6A methyltransferase, RNA methylation writer methyltransferase like 14 (METTL14), in T cells would trigger spontaneous colitis in mice, which is characterized by the Th1/Th17 phenotype. Treg (regulatory T cell) dysfunction leads to colitis development which depends on gut microbiota [12]. Similarly, mice with Foxp3-mediated deletion of the m6A writer protein methyltransferases like 3 (METTL3) in Tregs have been reported to trigger a serious systemic autoimmune response [13], which is one of the features of intestinal inflammatory conditions. Moreover, the new m6A-XPO1-NF-kB pathway activated in patients with Crohn’s Disease also clearly demonstrates that m6A plays a regulatory role in IBD [14].

In this study, m6A regulators differentially expressed in IBD samples were analyzed based on GSE87466 and GSE75214; METTL3 was selected given its key role in inflammation regulation [15–17]. The expression of METTL3 was determined in collected clinical IBD and normal control colon samples, as well as in the DSS-induced IBD mouse model. Mouse intestinal epithelial cells are widely used as in vitro models for IBD-related research [18, 19]. lipopolysaccharides (LPS) were known to be increased in IBD mucosa [20] and also use to mimic the inflammatory response in IBD [18, 21]. Herein, we transfected a mouse intestinal epithelial cell line MODE-K with lentivirus contained short hairpin RNA targeting METTL3 to achieve METTL3 knockdown, treated the cell line with LPS, and determined cell viability, apoptosis, apoptotic caspase3/9, inflammatory factor (IL-1β, TNF-α, IL-6, and IL-18) and inflammatory enzymes (COX-2 iNOS). METTL3 overexpression or knockdown effects on p65 phosphorylation were investigated in LPS-stimulated MODE-K cells. Co-effects of METTL3 overexpression and NF-κB inhibitor (JSH-23) on LPS-stimulated MODE-K cells were investigated. Lastly, the in vivo effects of METTL3 knockdown on DSS-induced IBD were investigated in the mouse model.

## Materials and methods

### m6A regulator selection

Through literature review, we collated a list of 19 m6A regulators from recently published literature, including 10 readers (IGF2BP1, IGF2BP2, IGF2BP3, METTL3, METTL14, WTAP, RBM15, RBM15B, ZC3H13, KIAA1429), 7 writers (YTHDC1, YTHDC2, HNRNPC, HNRNPA2B1, EIF3A, YTHDF2, YTHDF3), and 2 erasers (FTO and ALKBH5).

### Clinical sampling

A total of 15 cases ulcerative colitis tissues and 15 paired control tissues from distal end of colon ulcers were collected from patients with IBD undergoing resection surgery at the Second Xiangya Hospital after signing informed consents. The physician global assessment (PGA) was used to classify disease activity, based on medical history, physical examination, laboratory findings, and endoscopic examination [22]. Newly diagnosed and untreated patients that had no other inflammatory or infectious disease, and post colonoscopies, patients with IBD were included. The biopsies were snap frozen in liquid nitrogen or fixed in formalin. Frozen biopsies were stored at −80 °C until further analyses. The whole study was approved by the Ethics Committee of The Second Xiangya Hospital of Central South University (approval number: 2017-S117).

### qRT-PCR

Total RNA was isolated from cells and tissues using a RNeasy Mini Kit (Qiagen, Germantown, MD, USA) or TRIzol (Invitrogen Life Technologies, Carlsbad, CA, USA) according to the manufacturer’s instructions, Complementary DNA was synthesized from the extracted RNA by the StarScript II First-strand cDNA Synthesis Mix (GenStar, China) The expression levels of genes were examined using a SYBR^®^ Green PCR Master Mix (Qiagen GmbH) and An ABI7500 real-time PCR detection system (Applied Biosystems; USA). GAPDH expression was used as an internal reference. The relative expression levels were calculated using the 2^**−**ΔΔCt^ method The primer sequences for qRT-PCR were listed in Table S1.

### Immunoblotting

Protein samples were obtained from cells and tissue samples and the concentration was determined using a BCA Protein Assay Kit (Beyotime, China). Protein samples were then electrophoresed on 10–14% SDS-PAGE and transferred to the PVDF membrane. The membrane was blocked for 1 h at room temperature in Odyssey blocking buffer (LI-COR Bioscience, Lincoln, NE, USA) followed by overnight incubation with primary antibody at 4 °C. The primary antibodies were against METTL3 (15073-AP, Proteintech, USA), TNF-α (60291-1-Ig, Proteintech), iNOS (18985-1-AP, Proteintech), IL-6 (CSB-PA06757A0RB, CUSABIO, China), cleaved-caspase3 (ab34287, Abcam, USA), cleaved-caspase9 (10380-1-AP, Proteintech), IL-1β (12242, Cell signaling technology, USA), IL-18 (60070-1-Ig, Preoteintech), COX-2 (12375-1-AP, Proteintech), p-p65 (ab76302, Abcam), p65 (10745-1-AP, Proteintech) and GAPDH (endogenous control, 60004-1-Ig, Proteintech). Primary antibody detection was performed with a horseradish peroxidase-linked goat anti-rabbit immunoglobulin G (IgG) (SC-1004; Santa Cruz Biotechnology, Dallas, TX), or horse anti-mouse IgG (7076S; Cell Signaling Technology, Danvers, MA, USA), visualized with the Enhanced Chemiluminescence (ECL) detection system (Promega, Madison, WI, USA).

### Experimental mice

A total of 60 female C57BL/6 mice aged 6–8 weeks purchased from the SLAC experimental animal center (Changsha, China), and were housed in the animal facilities with a 12:12-h light/dark cycle, controlled temperature (22–24 °C), and humidity (50–60%), for 1-week quarantine with free access to water and food. All procedures were approved by The Animal Care and Use Committee of Central South University.

### Establishment and identify of DSS-induced colitis model in mice

Mice were randomly divided into differernt groups. Each mouse received 7 days of DSS treatment consisting of 7 days with 3% DSS in the drinking water. The control group animals were administered distilled water. The mice were monitored daily for survival and body weight. On day 8, mice were anesthetized and sacrificed for colon tissue harvesting. The harvested colon tissues were collected for immunoblotting and histological analysis. For histological analysis, colonic tissues were fixed in 4% paraformaldehyde and embedded in paraffin. Fixed tissues were cut into 5-mm-thick sections, placed on glass slides, and deparaffinized. The sections were stained with hematoxylin and eosin (H&E) and observed under a light microscope.

For lentivirus transfection, the lentivirus package system containing sh-NC or sh-METTL3 was transduced to HEK293FT cells and lentivirus was harvested by PEG precipitation. For in vivo studies, DSS-stimulated mice were randomly divided into sh-NC and sh-METTL3 groups and 50 μl of lentivirus (1 × 10^8^ TU) was delivered by intracolonic injection three times (at day 0, 2, and 4 during DSS treatment). H&E staining, immunoblotting analysis and body weight determination were performed as above-described.

For METTL3 inhibition, mice were intraperitoneal injection STM2457 (50 mg/kg, Selleck, China) or vehicle (same amount of DMSO) once daily for 7 days. H&E staining, immunoblotting analysis and body weight determination were performed as above-described.

### Histopathological analyses and immunohistochemistry (IHC) staining

The mice were euthanized under anesthesia and colon tissues were collected. Tissue samples were fixed by immersion in 10% buffered formaldehyde overnight, embedded in paraffin, and cut into 4-μm-thick sections. After deparaffinization, slides were stained with hematoxylin and eosin (H&E) or Masson’s trichrome staining by standard methods.

IHC examination was performed as described previously [23, 24]. Tissue sections were rehydrated, and endogenous peroxidases were quenched with 3% hydrogen peroxide. Slides were then incubated with primary antibodies against METTL3 or a rabbit IgG (isotype control, ab37415, abcam) overnight at 4 °C. After washing with PBS, the sections were incubated with poly-IgG-HRP antibody (Boster, Wuhan, China) for 30 min at 37 °C. The section was stained by a diaminobenzidine (DAB) kit (Boster) for 10 min and visualized under a microscope (Olympus, Kyoto, Japan). The histological results were evaluated by two pathologists in a blind manner.

### Assessment of disease activity index (DAI) score assessing disease severity

To assess the severity of colitis, the body weight, stool consistency, and blood in the stool were determined according to the previously published grading system [25]. Briefly, weight loss was scored as follows: score 0, none; score 1, 1–5%; score 2, 5–10%; score 3, 10–20%; score 4, >20%. Diarrhea was scored as follows: score 0, normal; score 2, loose stools; score 4, watery diarrhea. Blood in stool was scored as follows: score 0, normal; score 2, slight bleeding; score 4, gross bleeding.

### Assay of myeloperoxidase activity (MPO)

A MPO activity kit (Jiancheng Biotech, Nanjing) was used for MPO activity determination. Colon tissues (50 mg) were washed, homogenized in cooled phosphate-buffered saline (PBS, w/w 1:19). The total protein was determined with a bicinchoninic acid (BCA) assay kit (Beyotime). Then, the MPO activity was determined following the instruction of the kit. The absorbance of the resulting mixture was measured at 460 nm with a UV spectrophotometer (Shimadzu Corporation, Kyoto, Japan).

### Assay of nitric oxide (NO)

The nitric oxide (NO) content was calculated by measuring its stable metabolites, nitrite (NO2−) and nitrate (NO3−). Colon tissues (50 mg) were washed, homogenized in cooled phosphate buffered saline (PBS, w/w 1:9). The total protein was determined with a bicinchoninic acid (BCA) assay kit (Beyotime). Then, the NO level was determined following a method described by NO assay kit’s instruction (Jiancheng Biotech). The absorbance of the resulting mixture was measured at 550 nm with a UV spectrophotometer (Shimadzu Corporation).

### Assay of Malondialdehyde (MDA) levels

MDA was determined following a method described in the instruction of MDA assay kit ((Jiancheng Biotech). Colon tissue (100 mg) was washed, and homogenized in cooled PBS (PBS, w/w 1:9). The total protein was determined with a bicinchoninic acid (BCA) assay kit (Beyotime). The level of MDA was measured at 535 nm using a spectrophotometer.

### Cell lineage

Mouse intestinal epithelial cell line, MODE-K, was purchased from Shanghai GuanDao Biological Engineering (Shanghai, China) and cultured in Eagle’s Minimum Essential Medium supplemented with 10% FBS (Invitrogen, Carlsbad, CA, USA), 2 mM L-glutamine, 100 IU penicillin, and 100 mg/ml streptomycin at 37 °C in a humidified incubator containing 5% CO_2_.

### Cell transfection and treatment

For METTL3 knockdown or overexpression, lentivirus containing specific short hairpin RNA (#1 sh-METTL3 or #2 sh-METTL3, GenePharma, Shanghai, China) or METTL3-overexpressing fragment was transfected into MODE-K cells using polybrene (Invitrogen). 48 h later, the cells were harvested for further investigation. The scramble shRNA was used as a negative control (sh-NC). The sequences were listed in Table S1.

For LPS stimulation, MODE-K cells were incubated with 200 ng/ml LPS (Sigma-Aldrich, USA) for 48 h.

For NF-κB inhibition, MODE-K cells were incubated with 10 μM JSH-23 (Sigma-Aldrich) for 48 h.

### CCK-8 assay detecting cell viability

Cell viability was detected by Cell Counting Kit-8 (CCK-8, Beyotime). Cells were seeded in a 96-well plate for 24 h, after different treatments for 24, 48, and 72 h, the cells were added with 10 μL of CCK-8 solution, and incubated in an incubator at 37 °C for 3 h. The absorbance at 450 nm (OD450) was detected by a microplate reader (Bio-Rad, Hercules, USA).

### Flow cytometry detecting cell apoptosis

Following transfection and/or treatment, cells were collected and digested with trypsin (trypsin) without ethylenediaminetetraacetic acid at room temperature for 1 min. The digestion was terminated by adding DMEM (Corning) containing 10% FBS. The cells were centrifuged at 1000 × *g* at room temperature for 3 min and the supernatant was removed. The cells were washed twice with pre-cooled PBS and resuspended in 1× Annexin V binding buffer. Following the protocols of the Annexin V-FITC cell apoptosis detection kit (KeyGen Biotech), cells were stained with 5 µl Annexin V-FITC and 5 µl propidium iodide (PI) and measured by flow cytometry (Novocyte, Agilent, USA). The apoptosis rate is a sum of early apoptosis (Annexin V positive/PI negative) and late apoptosis (Annexin V positive/ PI positive).

### Statistical analysis

Data are processed using SPSS17.0 statistical software and presented as the mean ± S.D. of results from at least three independent experiments. Kruskal–Wallis test was used for non-parametric statistical analysis. A Student’s *t* test (two-tails) was used for statistical comparison between means where applicable. Differences among more than two groups in the above assays were estimated using one-way ANOVA. *P < 0.05; **P < 0.01.

## Results

### METTL3 is up-regulated in IBD and DSS-induced IBD model in mice

**Fig. 1:**
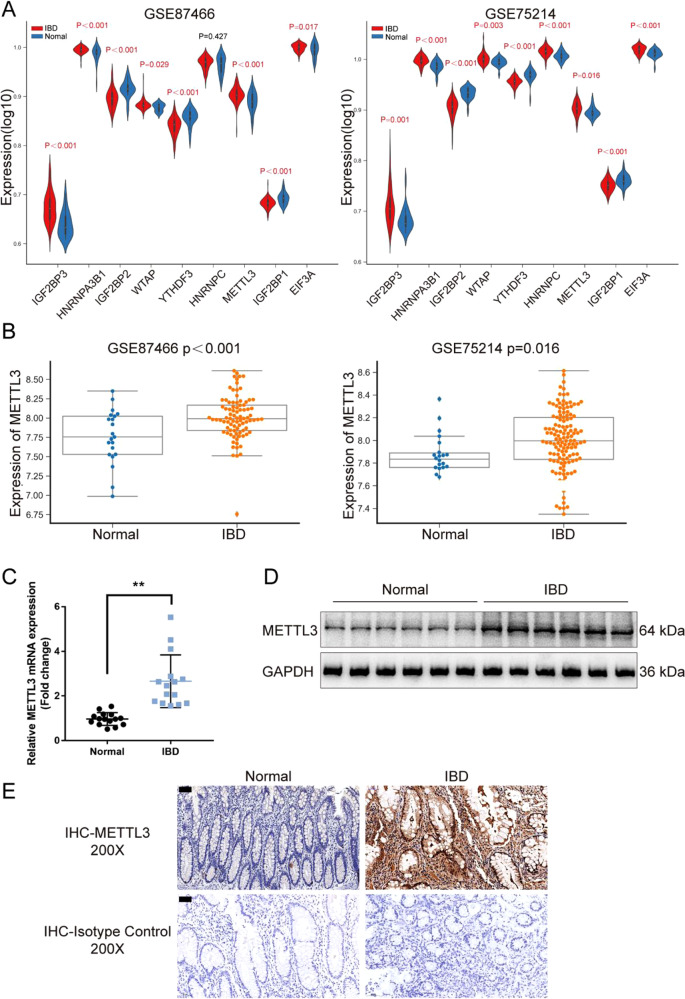
METTL3 is up-regulated in inflammatory bowel disease (IBD). **A** m6A regulators differentially expressed between IBD and normal samples were analyzed based on GSE87466 and GSE75214; the expression of 9 m6A core genes with significant differences (*P* < 0.05), including WTAP, METTL3, IGF2BP3, HNRNPC, HNRNPA2B1, EIF3A, IGF2BP2, YTHDF3, and IGF2BP1 was shown. **B** The expression of METTL3 in IBD and normal samples according to GSE87466 and GSE75214. **C** METTL3 mRNA expression was examined in collected IBD (*n* = 15) and normal samples (*n* = 15) using qRT-PCR. **D** METTL3 protein levels were examined in collected IBD (*n* = 15) and paired normal samples (*n* = 15) using immunoblotting. **E** METTL3 levels and distribution were examined in IBD and normal samples (*n* = 15) using immunohistochemical (IHC) staining. The isotype IgG controls were shown in the below panel. **p < 0.01, compared with normal group.

For identifying m6A regulators differentially expressed between IBD and normal samples, datasets GSE87466 and GSE75214 were downloaded and analyzed. A total of 9 m6A core genes with significant differences (*P* < 0.05) were obtained, including 6 up-regulated genes (WTAP, METTL3, IGF2BP3, HNRNPC, HNRNPA2B1, EIF3A) and 3 down-regulated genes (IGF2BP2, YTHDF3, IGF2BP1) (Fig. 1A). Among these regulators, METTL3 has a critical role in inflammation [15–17]. The expression of METTL3 was significantly up-regulated in IBD samples according to GSE87466 and GSE75214 (Fig. 1B). Consistently, in collected clinical IBD samples, METTL3 mRNA expression was significantly up-regulated (Fig. 1C) and METTL3 protein levels were increased (Fig. 1D) compared with that in normal samples. IHC staining also revealed increased METTL3 levels in IBD tissue samples (Fig. 1E). Moreover, in the DSS-induced IBD mouse model, the METTL3 expression was also examined. Fig. S1A shows that the colon length of mice in DSS-induced IBD was markedly shortened. The basal body weight of IBD mice decreased and the DAI score of IBD mice increased (Fig. S1B, C). Fig. S1D showed that DSS stimulation led to extensive colon tissue injury, such as crypt distortion and crypt atrophy, epithelial cell necrosis, and edema, neutrophil infiltration, and accumulation of lymphocytes between crypt basement and mucous muscle. DSS stimulation significantly increased NO, MDA, and MPO levels and increased expression of TNF-α, iNOS, and IL-6 in mice’s colon tissues (Fig. S1E–G). Consistent with clinical samples, METTL3 mRNA and protein expression showed to be markedly elevated within DSS-induced IBD mice (Fig. S1H, I). The up-regulation of METTL3 in IBD suggests its potential role in IBD etiology.

### METTL3 knockdown relives LPS-induced cellular inflammation and NF-κB activation in mouse intestinal epithelial cells

**Fig. 2:**
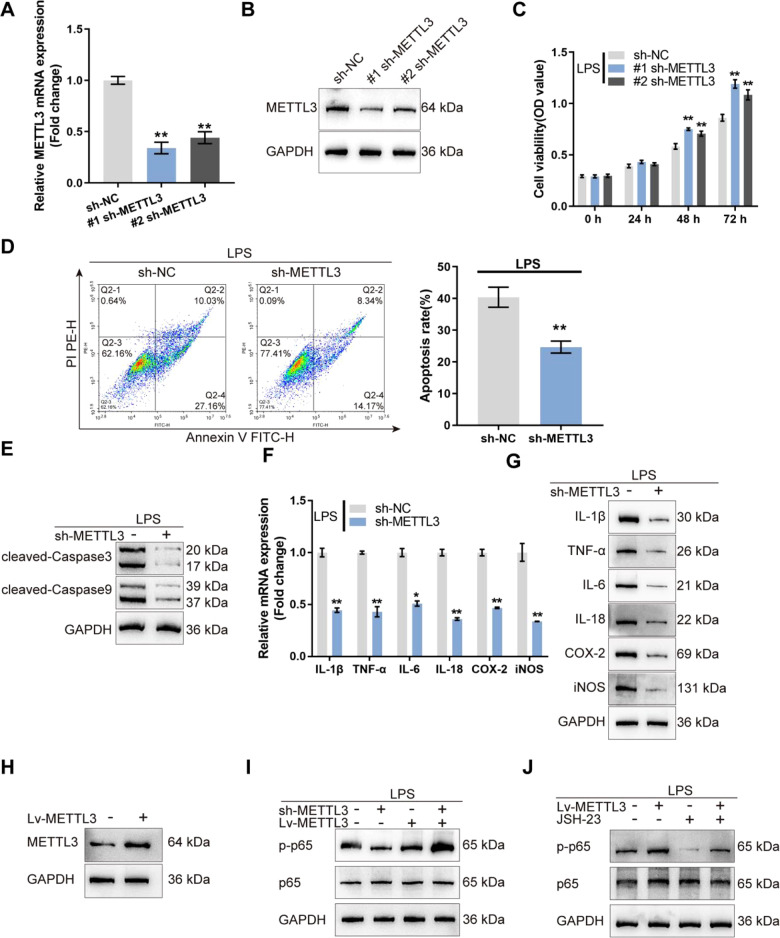
METTL3 knockdown relives LPS-induced cellular inflammation and NF-κB activation in mouse intestinal epithelial cells. **A**, **B** METTL3 knockdown was achieved in mouse intestinal epithelial cell line MODE-K by transfecting lentivirus containing specific short hairpin RNA (#1 sh-METTL3 or #2 sh-METTL3) and confirmed using qRT-PCR and immunoblotting. **C** MODE-K cells were transduced with #1 sh-METTL3 or #2 sh-METTL3 and examined for cell viability by CCK-8 assay; #1 sh-METTL3 was selected for further experiments. **D** MODE-K cells were transfected with sh-METTL3 and examined for cell apoptosis by flow cytometry. **E** The protein levels of cleaved-caspase3 and cleaved-caspase9 by immunoblotting. **F** The mRNA expression of IL-1β, TNF-α, IL-6, IL-18, COX-2, and iNOS by qRT-PCR. **G** The protein levels of IL-1β, TNF-α, IL-6, IL-18, COX-2, and iNOS by immunoblotting. **H** MODE-K cells were transfected with lentivirus-overexpressing METTL3 (Lv-METTL3) and examined for the protein levels of METTL3 using immunoblotting. **I** MODE-K cells were transfected with Lv-METTL3 or sh-METTL3, stimulated with LPS, and examined for the protein levels of p-p65 and p65 using immunoblotting. **J** MODE-K cells were transfected with Lv-METTL3, stimulated with LPS or co-stimulated with LPS and JSH-23 (NF-κB inhibitor), and examined for the protein levels of p-p65 and p65 using immunoblotting. *N* = 3, **p < 0.01, compared with sh-NC group.

Since METTL3 is up-regulated in IBD, METTL3 knockdown was achieved in mouse intestinal epithelial cell line MODE-K via transfecting lentivirus containing specific short hairpin RNA (#1 sh-METTL3 or #2 sh-METTL3) and confirmed using qRT-PCR and Immunoblotting (Fig. 2A, B). In LPS absent or stimulated MODE-K cells transfected with #1 sh-METTL3 or #2 sh-METTL3, cell viability was promoted, more promoted by #1 sh-METTL3 (Fig. 2C and Fig. S2A); #1 sh-METTL3 was selected for further experiments. Moreover, in LPS-stimulated MODE-K cells transfected with sh-METTL3, cell apoptosis was inhibited (Fig. 2D). Consistently, cleaved-caspase3 and cleaved-caspase9 protein contents showed to be remarkably reduced within sh-METTL3-transfected MODE-K cells (Fig. 2E). Regarding the inflammation, IL-1β, TNF-α, IL-6, IL-18, COX-2, and iNOS mRNA and protein expression were decreased by sh-METTL3 transfection (Fig. 2F, G). Thus, METTL3 knockdown improves LPS-induced cellular inflammation in MODE-K cells.

Given the central effect of the NF-κB signaling on LPS-caused inflammation [26, 27], the effects of METTL3 upon the NF-κB signaling were investigated. Firstly, we determined the expression of METTL3, p-p65, and p65 in response to LPS stimulation and found that LPS significantly increased METTL3 and p-p65/p65 levels (Fig. S2B, C). We transfected MODE-K cells with lentivirus-overexpressing METTL3 (Lv-METTL3), stimulated these cells with LPS, and determined METTL3 protein contents; under LPS stimulation, Lv-METTL3 transfection significantly increased METTL3 protein levels (Fig. 2H). In LPS-stimulated MODE-K cells, METTL3 overexpression significantly increased, whereas METTL3 knockdown decreased p-p65/p65 ratio (Fig. 2I). Subsequently, we transfected MODE-K cells with Lv-METTL3, stimulated these cells with LPS or co-stimulated with LPS and JSH-23 (NF-κB inhibitor), and determined p65 phosphorylation. Under LPS stimulation, METTL3 overexpression promoted, while JSH-23 stimulation inhibited p65 phosphorylation; JSH-23 partially attenuated the effects of METTL3 overexpression (Fig. 2J). Thus, under LPS stimulation, METTL3 could modulate NF-κB signaling activation.

### METTL3 affects LPS-induced cellular inflammation through the NF-κB signaling in MODE-K cells

**Fig. 3:**
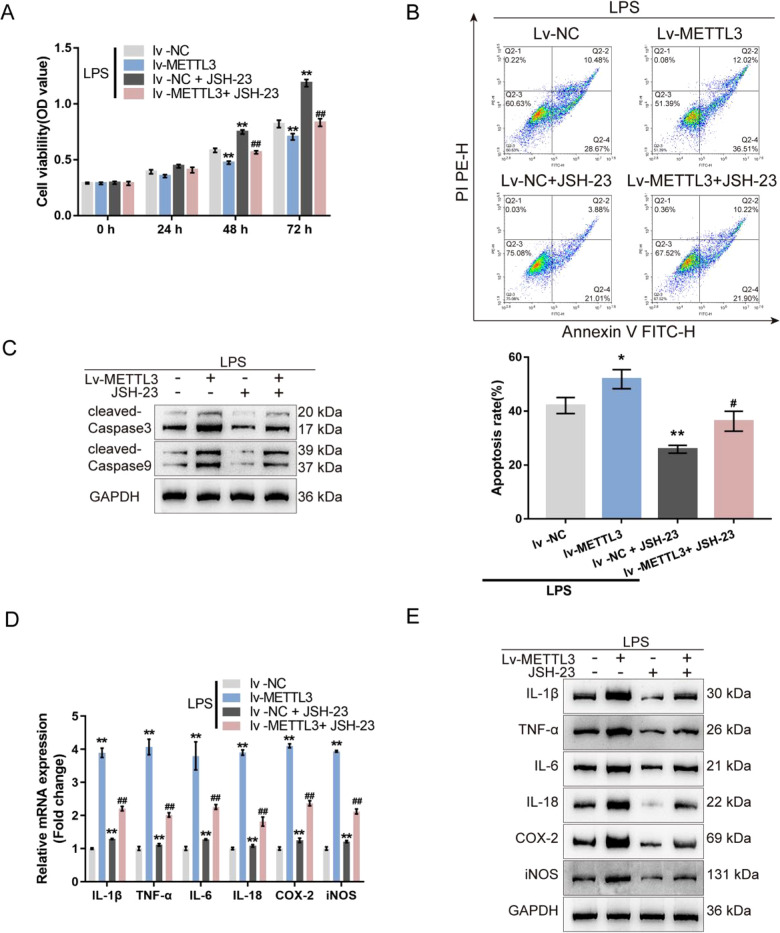
METTL3 affects LPS-induced cellular inflammation through the NF-κB signaling in MODE-K cells. MODE-K cells were transfected with Lv-METTL3, stimulated with LPS or co-stimulated with LPS and JSH-23 (NF-κB inhibitor), and examined for cell viability by CCK-8 assay (**A**); cell apoptosis by Flow cytometry (**B**); the protein levels of cleaved-caspase3 and cleaved-caspase9 by immunoblotting (**C**); the mRNA expression of IL-1β, TNF-α, IL-6, IL-18, COX-2, and iNOS by qRT-PCR (**D**); the protein levels of IL-1β, TNF-α, IL-6, IL-18, COX-2, and iNOS by immunoblotting (**E**). *N* = 3, **p < 0.01, compared with lv-NC group; ^##^*p* < 0.01, compared with lv-NC+JSH-23 group.

Secondly, the co-effects of METTL3 and the NF-κB pathway on LPS-treated MODE-K cells were investigated. without LPS stimulation, METTL3 overexpression also inhibits MODE-K cell viability which could be reversed by JSH-23 treatment (Fig. S2D). Under LPS stimulation, METTL3 overexpression suppressed cell viability and promoted cell apoptosis, while JSH-23 exerted opposite effects; the effects of METTL3 overexpression on MODE-K cell phenotypes were partially reversed by JSH-23 treatment (Fig. 3A, B). Consistently, METTL3 overexpression promoted, whereas JSH-23 treatment inhibited the cleavage of caspase3 and caspase9; the effects of METTL3 overexpression on caspase3 and caspase9 cleavage were partially abolished by JSH-23 treatment (Fig. 3C). Regarding cellular inflammation, METTL3 overexpression significantly up-regulated IL-1β, TNF-α, IL-6, IL-18, COX-2, and iNOS mRNA and protein expression, whereas JSH-23 treatment exerted opposite effects on these factors; similarly, METTL3 overexpression effects on inflammatory factors were partially eliminated by JSH-23 treatment (Fig. 3D, E).

### METTL3 affects DSS-induced IBD model through the NF-κB signaling

**Fig. 4:**
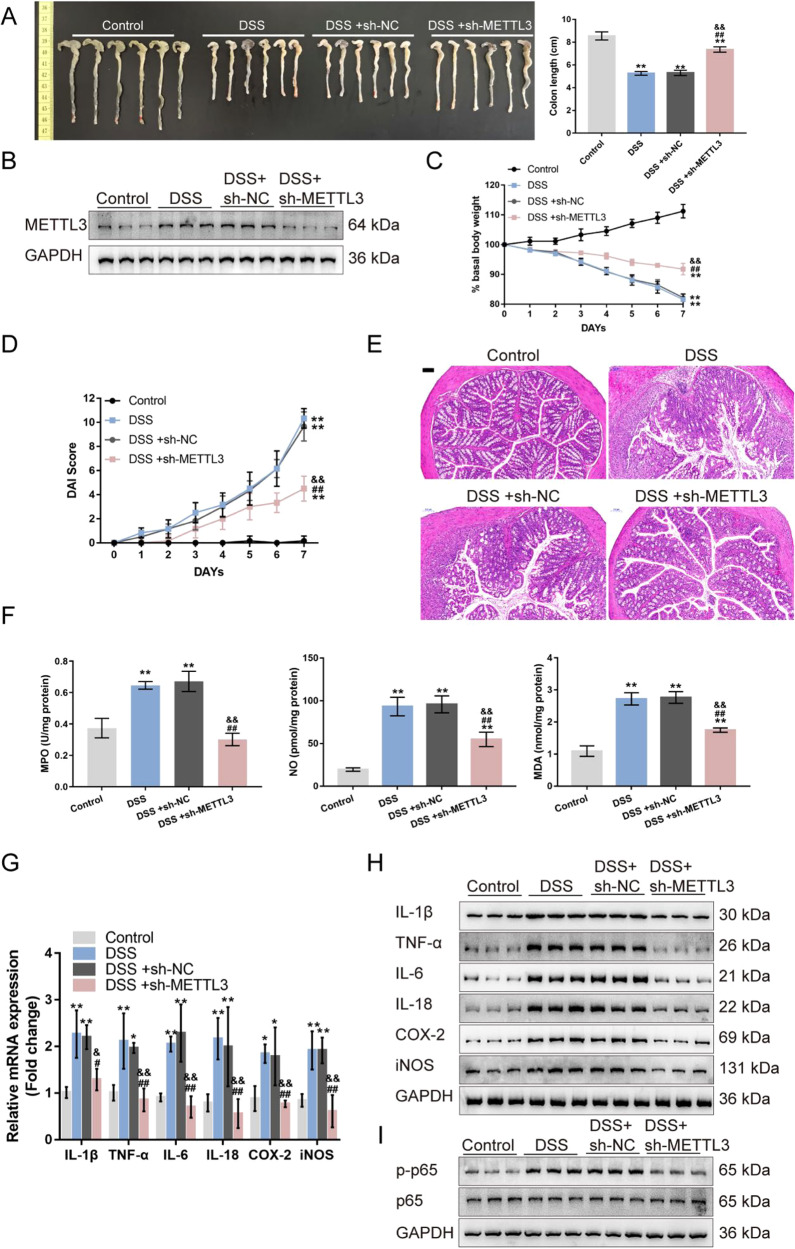
METTL3 knockdown affects DSS-induced IBD model through the NF-κB signaling. **A** Mice were divided into four groups: control, DSS-induced IBD model, DSS-induced mice received sh-NC injection, and DSS-induced mice received sh-METTL3 injection. DSS-induced IBD model and sh-NC/sh-METTL3 administration were conducted as described in the Materials and methods section. Colon length of mice in each group was determined. **B** The protein levels of METTL3 in colon tissues were examined using Immunoblotting. **C**–**D** Basal body weight and the disease activity index (DAI) score were examined on day 0, to 7. **E** At the end of the modeling, mice were sacrificed and the histopathological features of mice colon were examined using H&E staining. **F** Nitric oxide (NO), malondialdehyde (MDA), and myeloperoxidase activity (MPO) in colon tissues were examined. **G** The mRNA expression of IL-1β, TNF-α, iNOS, IL-6, IL-18, and COX2 were examined in colon tissues using qRT-PCR. **H** The protein levels of METTL3, IL-1β, TNF-α, iNOS, IL-6, IL-18, and COX2 in colon tissues were examined using Immunoblotting. **I** The protein levels of p-p65 and p65 were examined in colon tissues using Immunoblotting. *N* = 6, **p < 0.01, compared with control group; ^&&^*p* < 0.01, compared with DSS group; ^##^*p* < 0.01, compared with DSS+sh-NC group.

Lastly, the in vivo effects of METTL3 overexpression on DSS-induced IBD and the NF-κB signaling were investigated. We assigned mice into four groups: control, DSS-induced IBD model, DSS-induced mice received the sh-NC injection, and DSS-induced mice received the sh-METTL3 injection. DSS-induced IBD model and sh-NC/sh-METTL3 administration were conducted as described in the M&M section. DSS treatment significantly reduced, whereas METTL3 knockdown increased the colon length in DSS-induced IBD mice (Fig. 4A). The METTL3 expression was also increased by DSS treatment and successfully reduced by METTL3 knockdown in mice colon tissues (Fig. 4B). DSS-induced decreases in basal body weight and increases in the DAI score were partially reversed through METTL3 knockdown (Fig. 4C, D). At the end of the modeling, H&E staining revealed that DSS-induced histopathological changes were partially improved by METTL3 knockdown (Fig. 4E). Within DSS-caused IBD mice, NO, MDA, and MPO were significantly increased, whereas METTL3 knockdown partially decreased NO, MDA, and MPO levels (Fig. 4F). Regarding inflammation, METTL3 knockdown down-regulated IL-1β, TNF-α, iNOS, IL-6, IL-18, and COX2 mRNA expression and protein levels increased by DSS (Fig.6G-H). Regarding the NF-κB signaling pathway, DSS treatment promoted, whereas METTL3 knockdown inhibited the phosphorylation of p65 induced by DSS treatment (Fig. 4I).

**Fig. 5:**
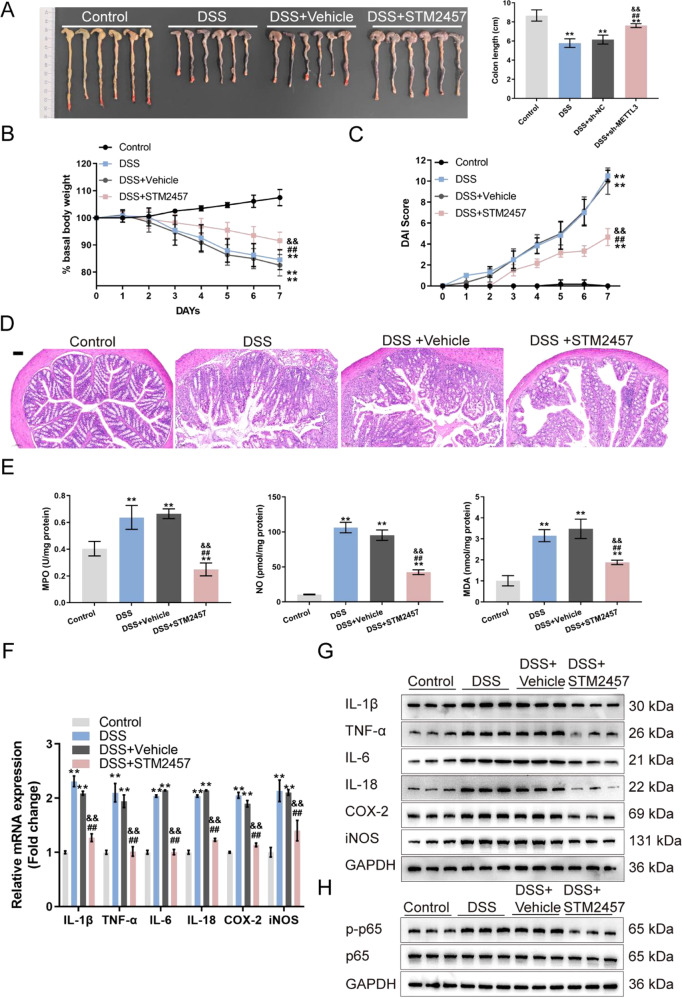
Inhibition of METTL3 affects DSS-induced IBD through the NF-κB signaling. **A** Mice were divided into four groups: control, DSS-induced IBD model, DSS-induced mice received vehicle control, and DSS-induced mice received STM2457 administration. DSS-induced IBD model and vehicle/STM2457 administration were conducted as described in the Materials and methods section. The colon length of mice in each group was determined. **B**, **C** Basal body weight and the DAI score were examined on day 0 to 7. **D** At the end of the modeling, mice were sacrificed and the histopathological features of mice colon were examined using H&E staining. **E** NO, MDA and MPO levels in colon tissues were examined. **F** The mRNA expression of IL-1β, TNF-α, iNOS, IL-6, IL-18, and COX2 were examined in colon tissues using qRT-PCR. **G** The protein levels of IL-1β, TNF-α, iNOS, IL-6, IL-18, and COX2 in colon tissues were examined using Immunoblotting. **H** The protein levels of p-p65 and p65 were examined in colon tissues using Immunoblotting. *N* = 6, **p < 0.01, compared with control group; ^&&^*p* < 0.01, compared with DSS group; ^##^*p* < 0.01, compared with DSS + vehicle group.

In addition, the METTL3 inhibitor STM2457 was used to further confirm the effect of METTL3 on the IBD mouse model. Mice were divided into four groups: control, DSS-induced IBD model, DSS-induced mice received vehicle control intraperitoneal injection, and DSS-induced mice received STM2457 intraperitoneal injection. Similar to METTL3 knockdown, STM2457 effectively increased colon length and body weight, reduced DAI score and improved the histopathological changes in DSS-induced IBD mice (Fig. 5A-D). DSS-induced up-regulation of NO, MDA, MPO, IL-1β, TNF-α, iNOS, IL-6, IL-18, and COX2 levels was also reduced by STM2457 (Fig. 5E-H).

## Discussion

In this study, METTL3, which plays a key role in inflammation regulation [15–17], has been recognized significantly up-regulated in IBD samples, DSS-induced IBD mice, and LPS-treated MODE-K cells. Within LPS-treated MODE-K cells, METTL3 knockdown promoted cell viability, inhibited cell apoptosis, decreased apoptotic caspase3/9 cleavage, and decreased the levels of proinflammatory cytokines (IL-1β, TNF-α, IL-6, and IL-18) and inflammatory enzymes (COX-2 and iNOS). Under the same conditions, METTL3 knockdown inhibited, whereas METTL3 overexpression promoted p65 phosphorylation in MODE-K cells; NF-κB inhibitor JSH-23 partially abolished the promotive effects of METTL3 overexpression upon p65 phosphorylation. Consistently, the effects of METTL3 overexpression upon LPS-stimulated MODE-K cells were partially abolished by JSH-23. Lastly, METTL3 knockdown or inhibition in DSS-induced IBD mice significantly ameliorated DSS-induced IBD and inhibited DSS-induced p65 phosphorylation.

METTL3 has been recognized as a pivotal methyltransferase and essential to the performance of m6A modification [28]. The absence or overexpression of METTL3 would surely change the total m6A methylated level, directly affecting the decay and translation of mRNA and miRNA biogenesis, and subsequently resulting in human diseases. In this study, we first recognized the up-regulation of METTL3 in clinical IBD colon samples, DSS-induced IBD mice, and LPS-stimulated mouse intestinal epithelial cell line MODE-K cells. As mentioned above, IBD is inflammation of the digestive system. Notably, METTL3 has a critical role in inflammatory responses. In fibroblast-like synoviocytes, which exert a crucial effect on the occurrence and development of rheumatoid arthritis, METTL3 may promote fibroblast-like synoviocyte activation and inflammatory response via the NF-κB signaling pathway [29]. METTL3 exerts a positive regulatory effect on the expression of MYD88, a pivotal upstream regulator of NF-κB pathway, and induces the activation of NF-κB, an osteogenic inhibitor, thereby inhibiting the development of osteogenesis [30]. Given these previous and present findings, up-regulation of METTL3 in IBD suggests its potential role in IBD progression.

As mentioned above, dysregulated cytokine secretion and signal transduction mechanisms via intestinal epithelial cells are involved in IBD pathogenesis [7]. Reportedly, cell death mechanisms are related to IBD progression [31]. Intestinal epithelial cells from IBD patients showed increased rates of apoptosis [32]. In this study, in LPS-stimulated mouse intestinal epithelial cells, METTL3 knockdown enhanced cell viability and suppressed cell apoptosis, as well as decreased the cleavage of pro-apoptotic caspase3/9. Cytokines exert a vital effect on IBD pathogenesis. Inflammatory factors (IL-1β, TNF-α, IL-6, and IL-18) and inflammatory enzymes (COX-2 and iNOS) are dramatically up-regulated within IBD [33–36]. In this study, LPS-induced increases in IL-1β, TNF-α, IL-6, IL-18, COX-2, and iNOS were partially eliminated by METTL3 knockdown. Moreover, in the DSS-induced IBD mouse model, METTL3 knockdown partially ameliorated DSS-induced intestinal injury and inflammation, as manifested as increased body weight and decreased DAI scores, decreased MPO, NO, and MDA, and reduced IL-1β, TNF-α, IL-6, IL-18, COX-2, and iNOS. Thus, in vitro and in vivo findings indicate that METTL3 knockdown could ameliorate LPS-induced mouse intestinal epithelial cell inflammation and apoptosis and DSS-induced IBD.

Regarding the mechanism, METTL3 reportedly exerts its functions via regulating NF-κB signaling [29, 30]. Herein, under LPS stimulation, METTL3 overexpression promoted, whereas METTL3 knockdown inhibited p65 phosphorylation, suggesting that NF-κB signaling was involved in METTL3 functions. The mice spontaneously developed severe intestinal inflammation under intestinal epithelial cell (IEC)-specific inhibition of NF-κB synthesis via conditional deletion of NEMO (IEC-specific ablation of NF-κB essential modulator). NEMO-deficient intestinal epithelium within this model showed an increase in apoptosis rate, an impairment of intestinal epithelial barrier integrity, resulting in an enhancement in mucosal immune responses, induced by bacteria invasion [37]. Moreover, mice with an IEC-specific deletion of IKK-β exhibited a downregulation in the expression of the epithelial-cell-restricted cytokine thymic stromal lymphopoietin and failed to develop a pathogen-specific TH2 response, thereby exacerbating proinflammatory Th1 cytokine production following parasitic infection [38]. Consistently, METTL3 overexpression aggravated LPS-induced cellular inflammation in mouse intestinal epithelial cells, whereas NF-κB inhibitor JSH-23 significantly attenuated the effects of METTL3 overexpression. Similarly, METTL3 knockdown in DSS-induced IBD mice also inhibited p65 phosphorylation, suggesting METTL3 regulation of the NF-κB pathway in vivo.

In conclusion, METTL3 overexpression aggravates LPS-induced cellular inflammation in mouse intestinal epithelial cells and DSS-induced IBD in mice via activating the NF-κB signaling. Since IBD involves functional impairment of intestinal epithelial cells, concomitant with the infiltration of the lamina propria by inflammatory cells [39], the effects of METTL3 on immune cells in the regulation of intestinal inflammation remain to be investigated in our future study.

## Supplementary information

supplementary figure and table legends

FIG S1

FIG S2

Table S1

## Data Availability

All data generated or analyzed during this study are included in this published article and its supplementary information files.
