# Supplementary material for: METTL3 overexpression aggravates LPS-induced cellular inflammation in mouse intestinal epithelial cells and DSS-induced IBD in mice
Source: Cell Death Discov. 2022 Feb 14;8:62. doi: 10.1038/s41420-022-00849-1 (PMC8844074; doi:10.1038/s41420-022-00849-1)
Supplement: Supplementary file 1 — supplementary figure and table legends [file 41420_2022_849_MOESM1_ESM.docx]

**Fig.S1 METTL3 is up-regulated in the DSS-induced IBD model in mice. (A)** DSS-induced IBD model was established in mice. The colon length of mice in each group was determined. **(B-C)** Basal body weight and the DAI score were examined on day 0 to 7. **(D)** At the end of the modeling, mice were sacrificed and the histopathological features of mice colon were examined using H&E staining. **(E)** NO, MDA, and MPO in colon tissues were examined. **(F-G)** The mRNA expression and protein levels of TNF-α, iNOS, and IL-6 were examined in DSS-induced IBD and normal control colon tissues using qRT-PCR and immunoblotting. **(H)** METTL3 mRNA expression was examined in DSS treated and normal control colon tissues using qRT-PCR. **(I)** METTL3 levels and distribution were examined in IBD and normal colon tissues using IHC staining. The isotype IgG controls were shown in the below panel. N=6, ** p<0.01 compared to control group.

**Fig.S2 MODE-K cell viability and expression of METTL3, p65 and p-p65.** (A) METTL3 was knocked down in MODE-K cells, the cell viability was determined in absence of LPS stimulation. (B-C) The expression of METTL3, p65 and p-p65 in MODE-K cells under LPS stimulation was determined by immunoblotting. (D) MODE-K cells were overexpressed with METTL3 and treated with JSH-23, the cell viability was determined. N=3, ** p<0.01, compared with sh-NC or lv-NC group; ## p<0.01, compared with lv-NC+JSH-23 group.

Table S1 the primers sequence.
