# Supplementary figures and images for: METTL3 overexpression aggravates LPS-induced cellular inflammation in mouse intestinal epithelial cells and DSS-induced IBD in mice
Source: Cell Death Discov. 2022 Feb 14;8:62. doi: 10.1038/s41420-022-00849-1 (PMC8844074; doi:10.1038/s41420-022-00849-1)

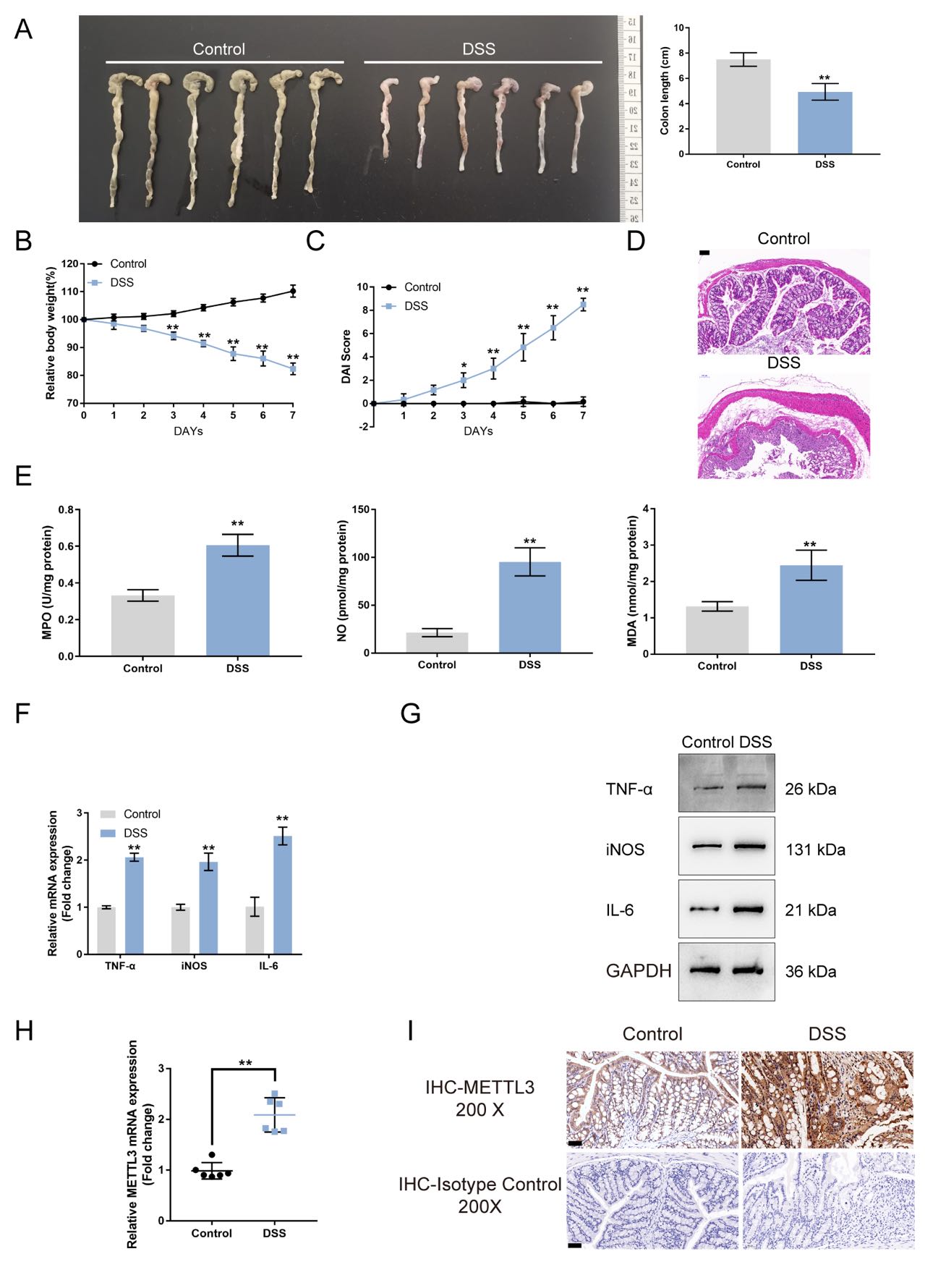

Supplement: Supplementary file 2 — FIG S1 [file 41420_2022_849_MOESM2_ESM.jpg]

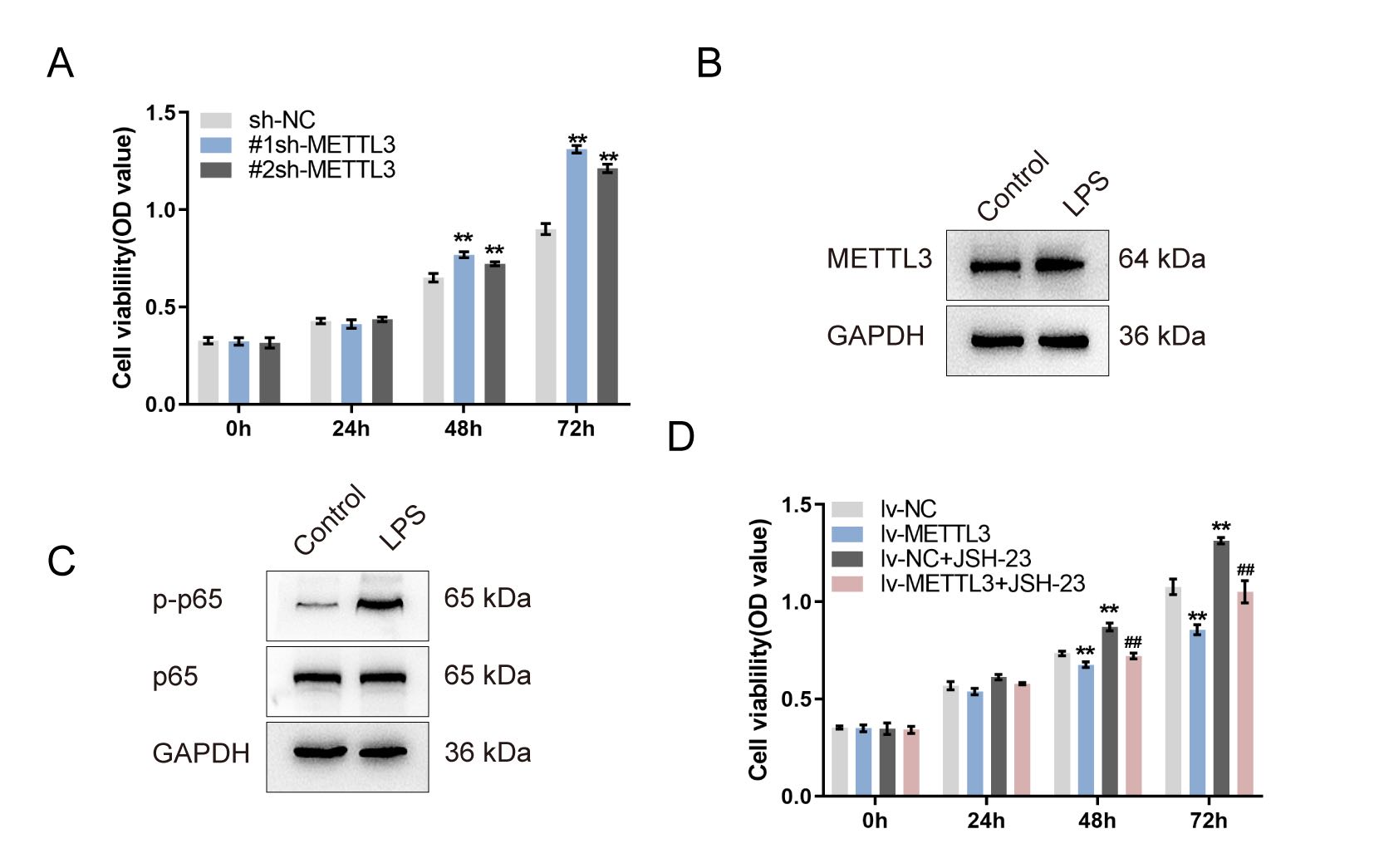

Supplement: Supplementary file 3 — FIG S2 [file 41420_2022_849_MOESM3_ESM.jpg]
