# Supplementary material for: METTL3 overexpression aggravates LPS-induced cellular inflammation in mouse intestinal epithelial cells and DSS-induced IBD in mice
Source: Cell Death Discov. 2022 Feb 14;8:62. doi: 10.1038/s41420-022-00849-1 (PMC8844074; doi:10.1038/s41420-022-00849-1)
Supplement: Supplementary file 4 — Table S1 [file 41420_2022_849_MOESM4_ESM.docx]

Table S1 the primers sequence

| RT-PCR  TNF-α | CCCTCACACTCAGATCATCTTCT | GCTACGACGTGGGCTACAG |
| --- | --- | --- |
| RT-PCR  iNOS | GTTCTCAGCCCAACAATACAAGA | GTGGACGGGTCGATGTCAC |
| RT-PCR  IL-6 | TAGTCCTTCCTACCCCAATTTCC | TTGGTCCTTAGCCACTCCTTC |
| RT-PCR  METTL3  human | CCAGCACAACATCTGTGGC | CGCTTTACCTCAATCAACTCCTG |
| RT-PCR  METTL3 | CATTGCCCACTGATGCTGTG | AGGCTTTCTACCCCATCTTGA |
| RT-PCR  COX-2 | TGAGCAACTATTCCAAACCAGC | GCACGTAGTCTTCGATCACTATC |
| RT-PCR  IL-18 | GACTCTTGCGTCAACTTCAAGG | CAGGCTGTCTTTTGTCAACGA |
| RT-PCR  IL-1β | GCAACTGTTCCTGAACTCAACT | ATCTTTTGGGGTCCGTCAACT |
| RT-PCR  GAPDH  human | ACAGCCTCAAGATCATCAGC | GGTCATGAGTCCTTCCACGAT |
| RT-PCR  GAPDH | AGGTCGGTGTGAACGGATTTG | TGTAGACCATGTAGTTGAGGTCA |
| Sh-NC | GATCCGCAGATGAAGGCACGGTCACGCTCGAGGCAGATGAAGGCACGGTCACGTTTTTG | AATTCAAAAAGCAGATGAAGGCACGGTCACGCTCGAGGCAGATGAAGGCACGGTCACGG |
| Sh-METTL3 1# | GATCCGCTGCACTTCAGACGAATTATCTCGAGATAATTCGTCTGAAGTGCAGCTTTTTG- | AATTCAAAAAGCTGCACTTCAGACGAATTATCTCGAGATAATTCGTCTGAAGTGCAGCG |
| Sh-METTL3 2# | GATCC GCTACAGGCCACACCTTAAGA CTCGAGTCTTAAGGTGTGGCCTGTAGC TTTTTG | AATTCAAAAAGCTACAGGCCACACCTTAAGACTCGAGTCTTAAGGTGTGGCCTGTAGC G |
| METTL3 overexpression  lentivirus | ctagcgtttaaacttaagcttATGTCGGACACGTGGAGCTC | tgctggatatctgcagaattcCTATAAATTCTTAGGTTTAGAGATGATGCC |
